# An analysis of the adolescents’ hazard perception when crossing road from the perspective of personality characteristics based on an eye-tracking study

**DOI:** 10.1371/journal.pone.0267309

**Published:** 2022-05-06

**Authors:** Ning Wang, Ruosong Chang, Fang Wu

**Affiliations:** 1 School of Psychology, Liaoning Normal University, Dalian, P.R. China; 2 Computer and Mathematics Basic Teaching Department, Shenyang Normal University, Shenyang, P.R. China; 3 The Second Affiliated Middle School of Liaoning Normal University, Dalian, P.R. China; Tongji University, CHINA

## Abstract

Using the Road Situation Video Test Paradigm and Eye-Tracking Analysis Method, according to the two-stage processing model of hazard perception (HP), this study divides HP into two stages: perception and behaviour response, and compares the different roles of sensation seeking level in two different processing stages of perception and behaviour response when adolescents are crossing the road. The results show that: (1) **In the perception stage**, adolescents with high sensation seeking, especially boys with high sensation seeking perceive danger faster than boys with low sensation seeking and girls, they are more aware of danger. Particularly, boys with high sensation seeking show a stronger advantage of attention processing to hazard in the early stage of perception processing from 8^th^ and 9^th^ grades. (2) **In the behaviour response stage**, adolescents with high sensation seeking, especially the boys are less sensitive, have stricter judgment criteria, and are more likely to make dangerous decisions when crossing the road. Girls with different sensation seeking levels are more sensitive and more cautious when crossing the road, make the probability of judging dangerous situation is higher, so they choose a more conservative way, and may be less likely to across the road.

## Introduction

The Research Report on the Status of Road Traffic Injuries among Children in China (2014) [1] states that road traffic injuries are the second leading cause of death among children aged 5 to 14 and the leading cause of death among boys in this age group. 3,994 young children under the age of 18 died in road traffic accidents nationwide in 2013, with the 12 to 14 age group accounting for 11.99% of the total number of deaths, and walking is their leading mode of transport. The Programme for the Development of Chinese Children (2021–2031) [2] clearly states the requirement to "train children to develop good traffic behaviour".

From the perspective of cognitive development, HP is an important factor influencing the risk of pedestrian accidents [3]. Children aged under 14 are more easily to become victims of road traffic injuries because they are not fully mature in physical development, motor skills, cognitive control and emotional factors, and their HP ability is lower [4]. HP is an individual’s ability to recognise and respond to road hazards in a timely manner, and is a significant predictor of road risk behaviours [5]. A common measure of HP is the Road Situation Video Test (RSVT), which measures correct response rate, reaction time and eye-tracking fixation time [6]. In pedestrian HP measurement studies, it is generally accepted that faster and more correct reaction rates and shorter fixation times indicate better HP. According to signal detection theory, there are two indicators for measuring pedestrian traffic judgement response-sensitivity(*d*’) and judgement criterion (*β*). The higher the *d*’ value, the stronger the hazard discrimination, and the bigger the *β* value, the stricter the judgement criterion and the slower the reaction to potential hazards [7].

HP skills depend on the individuals’ cognitive abilities. Junior high school students are in the early and middle adolescent stages where they have not developed mature HP skills and experienced difficulties in determining the safe time to cross the road [8], thus behaviour not well when crossing road [9]. They focus only on distance factors and fail to use given information effectively to choose a safe route [10], lacking awareness of common hazards at certain road intersections, lacking visual search ability, having difficulties in perceiving and extracting selective attention and temporal information [11], cognitive skills to handle complex traffic situations that are still lower than those of adults [12]. All these factors are associated with their immature cognitive development.

HP is also conditioned by personality development. From a personality development perspective, children’s road traffic injuries are significantly and positively correlated with sensation seeking, and the higher the sensation seeking level in children, the more traffic risk-taking behaviours will occur and the more likely they are to suffer road traffic injuries [13]. Adolescence is a critical period for the development of sensation-seeking traits, and studies have found that adolescents aged 10 to 15 are at an increasing sensation seeking level [14]. There are more possibility of being at risk on the road than primary and high school students [15] because they are sensitive to novel stimuli, are willing to engage in challenging activities and are eager to feel the pleasure of stimulation. Some studies have found that high sensation seeking adolescents are more prone to traffic injuries [16], and studies on driver perception of danger have reached similar conclusions [17]. High sensation seekers may ignore traffic rules and speed limits and engage in more risky behaviours under impulsive situations. These may lead to accidents or conflicts [18]. Their driving style that pursues risk stimulus makes them take lightly the warning signals of other vehicles, which may reduce their hazard recognition.

Since adolescents are the pedestrians most likely to take risks, and junior high school students are in their puberty, which is the peak period of high development of sensation seeking level, so this study is limited to junior high school students. It is of great educational significance to assess adolescents’ perception of road crossing hazards and exploring the influence of sensation seeking for building safety awareness and developing safety skills for adolescents.

According to the two-stage processing model of HP [19], HP can be divided into two stages: perception and behaviour response. Does an individual’s sensation seeking level have the same effect on the two stages of HP? Is the effect of sensation seeking level present throughout the processing of perception stage, or does it only play a role in the behaviour response stage? For high sensation seeking adolescents, once a hazard appears on the road, the novel change is likely to be picked up by them first, so we hypothesise that high sensation seeking adolescents will be able to perceive changes on the road and recognise hazards more quickly, as evidenced by a shorter latency of first fixation and a longer duration of the first fixation (Hypothesis 1). However, high sensation seeking adolescents may also be less attentive to danger due to stimulus seeking, so we hypothesise that high sensation seeking adolescents have stricter judgement standards and lower sensitivity in the behaviour response stage (Hypothesis 2). Since the activation and arousal needs of sensation seeking peak around the age of 14 (junior high school year) [14], we hypothesise that the role of sensation seeking in influencing perception stage and behaviour response stage will be greater as the junior high school year progresses (Hypothesis 3). The differences in grade, gender and personality (sensation seeking traits) in adolescents’ HP explored in this study have important implications for their traffic safety awareness development and healthy personality development.

## Study 1: Effect of sensation seeking on adolescents’ perception stage when crossing road

### Materials and methods

#### Participants

A total of 99 students from junior high schools (grade: 7^th^, 8^th^ and 9^th^) in Dalian were enrolled, with an average age of 13.55±1.04 years, all right-handed and with normal or corrected vision. The students were asked to answer the questions in the questionnaire (See Appendix A in S1 File for details). The median method was used to screen out 56 high sensation seekers and 43 low sensation seekers, the median score is 36, including 47 Grade 7 students (26 boys and 21 girls, mean age of 12.79±0.61 years), 28 Grade 8 students (13 boys and 15 girls, mean age of 13.60±0.33 years) and 24 Grade 9 students (14 boys and 10 girls, mean age of 14.99± 0.61 years). None of the subjects had participated in a similar HP experiment prior to this experiment. All received the appropriate reward after the experiment.

### Research tools

*The Sensation Seeking Scale for Primary and Secondary School Students* developed by Chen Lina and Zhang Ming was used to screen high and low sensation seeking students from each grade and to serve as the experimental sample, with high internal consistency (Cronbach’s α = 0.877).

The Tobii T600 eye tracker was used to record the subjects’ eye movements at a sampling rate of 600Hz. The Tobii T600 allows participants to shake their heads within a certain range during the experiment. Participants can complete the test in a relatively natural state. The Tobii Pro Lab x64 was used to delineate the area of interest.

### Materials and procedure

Under good weather conditions, videos of real traffic situations were shot from the perspective of pedestrians crossing the road in and around Dalian city. The original videos were edited using video editing software.

17 boys and 14 girls (mean age of 13.55±0.65 years) were selected to rate the edited videos. The participants were required to report: the presence of danger in the video, the time when the danger appeared, whether the danger was common in daily life, the frequency of occurrence, whether the process of appearance and development of the danger was clearly defined, and the number of dangers appearing at the same time. According to the purpose of this study, and taking into account the results reported by the participants, videos with low frequency of occurrence, relatively vague definition of the appearance and development process of hazards, and more than one or one type of hazards appearing at the same time were deleted, and a total of 12 videos of traffic hazard situations were finally retained, with an average duration of 9.83±2.04 seconds. See Appendix B in S3 File for details.

Each subject entered the laboratory individually was calibrated and given the guiding language by the experimenter, and was formally put through 12 videos of real road traffic situations after practising 3 videos. In the video, the subject was assumed to be standing at the position of the arrow and was about to cross the road in the direction of the arrow. When the subject saw a hazard on the road that could cause harm to him, he immediately pressed a button and the current video disappeared immediately after the response and automatically jumped to the next video. During the experiment, the subject was asked to minimise physical movements and to prepare by placing the index finger of the right hand on the Enter key of the keyboard. The experimental environment was kept quiet and the light was soft and constant.

### Experimental design

A three-factor experimental design of 3 (grade: 7^th^, 8^th^ and 9^th^) x 2 (gender: male, female) x 2 (sensation seeking level: high, low) was adopted. Grade, gender and sensation seeking level were the between-subject variables. The dependent variables were two eye-tracking indicators: latency of first fixation, which refers to how long it took for the subject to first fixate on the area of interest, and first fixation duration, which refers to the duration of the subject’s first fixation in an area of interest.

### Area of interest(AOI)

**Fig 1:**
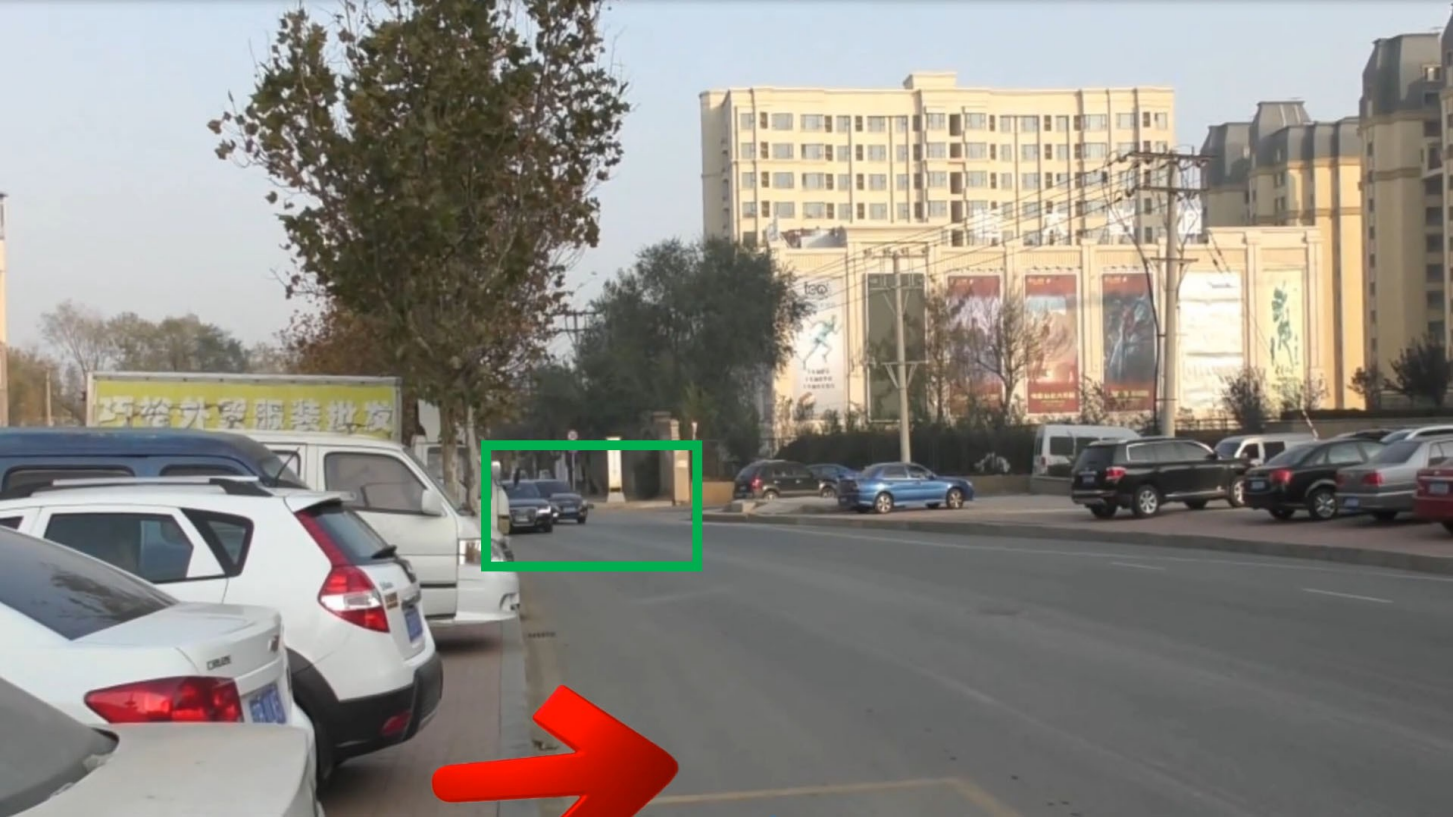
Dangerous vehicle AOI in different traffic scenarios. http://doi.org/10.6084/m9.figshare.19149749.

In this research, dangerous vehicles were used as eye-tracking AOI, and the entire scene area was 1918 x 1078 pixels, with each AOI being equaled in size, all 197 x 137 pixels, such as Fig 1.

### Statistical analysis

A multivariate ANOVA was performed on the eye-tracking data using SPSS 22.0.

### Results and analysis

#### Latency of first fixation

The statistical results of latency of first fixation of adolescents during road crossing are shown below (Table 1).

**Table 1:** Mean and standard deviation of latency of first fixation during road crossing for adolescents(s).

Grade	Male		Female	
	Low sensation seeking	High sensation seeking	Low sensation seeking	High sensation seeking
	(*N* = 24)	(*N* = 29)	(*N =* 19)	(*N =* 27)
Grade 7 (*N* = 47)	2.77±0.43	2.41±0.17	2.44±0.23	2.48±0.17
Grade 8 (*N* = 28)	2.73±0.31	2.36±0.20	2.56±0.40	2.44±0.14
Grade 9 (*N* = 24)	2.99±0.49	2.32±0.26	2.31±0.56	2.45±0.20

https://figshare.com/articles/figure/tabel1-4/19149803?file=34023125

The results of the multivariate ANOVA on latency of first fixation at road crossing of adolescents showed that the main effect of grade was not significant; the main effect of gender was significant, *F(*_1, 86)_ = 5.347, *p*<0.05, *η*^2^ = 0.059, with boys having a significantly longer latency of first fixation at road crossing than girls; the main effect of sensation seeking was significant, *F*_(1, 86)_ = 13.324, *p*<0.001, *η*^2^ = 0.134, with low sensation seeking adolescents having a significantly longer latency of first fixation at road crossing than high sensation seeking adolescents. The interaction between grade and gender was not significant; the interaction between grade and sensation seeking was not significant; and the interaction between gender and sensation seeking was significant, *F*_(1, 86)_ = 15.725, *p*<0.001, *η*^2^ = 0.155. Further simple effect analysis revealed that latency of first fixation across the road was significantly longer for low sensation seeking boys than for high sensation seeking boys, *t*_(1, 86)_ = 4.707, *p*<0.001, Cohen’s *d* = 0.75, but there was no significant difference in latency of first fixation across the road for girls with different levels of sensation seeking. Grade, gender and sensation seeking interactions were not significant. The covariate age was not significantly different.

#### Duration of first fixation

Statistics on the duration of first fixation of adolescents when crossing the road are shown below (Table 2).

**Table 2:** Mean and standard deviation of the duration of first fixation for adolescents crossing road (*s*).

Grade	Male		Female	
	Low sensation Seeking (*N* = 24)	High sensation seeking (*N* = 29)	Low sensation seeking (*N* = 19)	High sensation seeking (*N* = 27)
Grade 7 (*N* = 47)	0.33±0.10	0.32±0.10	0.30±0.09	0.34±0.08
Grade 8 (*N* = 28)	0.24±0.09	0.44±0.06	0.36±0.10	0.37±0.08
Grade 9 (*N* = 24)	0.25±0.08	0.33±0.09	0.43±0.06	0.36±0.11

https://figshare.com/articles/figure/tabel1-4/19149803?file=34023125

The results of the multivariate ANOVA on the duration of first fixation of adolescents when crossing road showed that: the main effect of grade was not significant; the main effect of gender was significant, *F*_(1, 86)_ = 4.602, *p*<0.05, *η*^2^ = 0.051, the duration of first fixation point of boys crossing road was significantly shorter than the duration of first fixation of girls crossing road; the main effect of sensation seeking was not significant; the interaction of grade and gender was not significant; the interaction of grade and sensation seeking was not significant; the interaction of gender and sensation seeking was significant, *F*_(1, 86)_ = 5.192, *p*<0.05, *η*^2^ = 0.057, the duration of first fixation of high sensation seeking boys was longer than that of low sensation seeking boys, while the duration of first fixation of girls did not differ significantly by sensation seeking level; the interaction between grade, gender and sensation seeking was significant, *F*_(2, 86)_ = 3.756, *p*<0.05, *η*^2^ = 0.080. A simple effect analysis showed that in 7^th^ grade, there was no significant difference in the duration of first fixation between sensation seeking groups for either boys or girls (*p*>.05); in 8^th^ grade, the duration of first fixation was significantly longer in the high sensation seeking boys than in the low sensation seeking boys (*p* < .05) and there was no difference in the girls’ group by sensation seeking level (*p*>.05); in 9^th^ grade, the first fixation duration was significantly longer in the high sensation seeking boys’ group than in the low sensation seeking boys’ group (*p* < .05) and in the low sensation seeking girls’ group than in the high sensation seeking girls’ group (*p* < .05). The covariate age did not differ significantly.

## Study 2: Effects of sensation seeking on adolescents’ behaviour response stage when crossing road

### Materials and methods

#### Participants

66 high sensation seekers and 55 low sensation seekers were screened (the same method as Study 1), including 54 7^th^ grade students (32 boys and 22 girls, mean age of 12.92±0.51 years), 39 8^th^ grade students (21 boys and 18 girls, mean age of 13.93±0.48 years) and 28 9^th^ grade students (16 boys and 12 girls, mean age of 15.20± 0.52 years). All participants had normal visual acuity or corrected visual acuity. None of the subjects had participated in a similar HP experiment prior to this experiment. All were paid accordingly after the experiment.

### Materials and procedure

Under good weather conditions, videos of real traffic situations were shot from the perspective of pedestrians crossing road in and around Dalian city. The original videos were edited using video editing software.

17 boys and 14 girls (mean age of 13.55±0.65 years) were selected to rate the edited videos. The participants were required to report whether there is danger in the video, and the finally retained traffic situation includes 20 videos of dangerous traffic situation and non-dangerous traffic situation, with an average duration of 10.20 ± 1.54 seconds, and the number of videos of the two situations is the same. See Appendix C in S5 File for details.

The experiment was conducted in a quiet and softly lit room. The subjects first filled out an informed consent form and a basic information questionnaire. Throughout the experiment, the computer was switched on silently, i.e. only the images of the traffic situation video were presented without sound.

The subject was first presented with the guiding language by the experimenter, and the experimenter explained it to him/her. During the experiment the subject was shown 20 videos of real road traffic situations in which the subject was assumed to be standing at the position of an arrow and was about to cross the road in the direction of the arrow. After the subject gave clear instructions, the experiment was started and the video was played randomly, after which the subject completed the behaviour choice task.

### Study design

A three-factor experimental design of 3 (grade: 7^th^, 8^th^ and 9^th^) x 2 (gender: male, female) x 2 (sensation seeking level: high, low) was used. Where grade, gender and sensation seeking level were all between-subject variables. The dependent variables were sensitivity *(d´*), which refers to the subject’s ability to discriminate between traffic hazards, the higher the *d´* value, the higher the subject’s sensitivity; and judgement standard (*β*), which refers to the subject’s criteria for judging a stimulus as "dangerous", the bigger the *β* value, the stricter the subject’s judgement standard, and the smaller the *β* value, the looser the subject’s judgement standard. Age is a covariate.

### Statistical analysis

A multivariate ANOVA was conducted using SPSS 22.0 for the *d´* and *β* values under the traffic scenario video respectively.

### Results and analysis

Sensitivity (*d′*) and judgement criteria (*β*) were calculated based on signal detection theory and the statistical methods of Stanislaw and Todorov (1999).

The sensitivity (*d′*) was calculated as follows:

*d′* = Z hit—Z false alarm

The judgement criterion (*β*) was calculated using the following formula:

*β =* O hit / O false alarm

A Hit means that in a dangerous situation, the subject reports "No", that is, when there is a danger, the subject finds danger and chooses not to cross road. A Miss means that in a dangerous situation, the subject reports "Yes", that is, when there is a danger, the subject does not find the danger and chooses to cross the road. A Correct Refusal (CR) means that in a safe situation, the subject reports "Yes", i.e., no danger is present, but the subject does not find the danger and chooses to cross the road. A False Alarm (FA) means that in a safe situation, the subject reports "No", i.e., no danger is present, but the subject finds the danger and chooses not to cross the road. See Tables 3 and 4 below for details.

**Table 3:** Signal detection theory indicators.

Situation	Subjects’ response	
	No (not cross the road)	Yes (cross the road)
Dangerous	Hit	Miss
Safe	FA	CR

https://figshare.com/articles/figure/tabel1-4/19149803?file=34023131

**Table 4:** Mean and standard deviations of sensitivity d and judgment criterion when crossing road for adolescents*(s)*.

Grade	Male				Female			
	Low sensation seeking		High sensation seeking		Low sensation seeking		High sensation seeking	
	(*N* = 38)		(*N* = 31)		(*N* = 17)		(*N* = 35)	
	*d´*	*β*	*d´*	*β*	*d´*	*β*	*d´*	*β*
Grade 7 (N = 54)	2.97±0.656	1.11±3.102	2.51±0.782	4.07±5.330	2.89±0.475	0.09±.013	2.86±0.780	3.40±5.449
Grade 8 (*N* = 39)	3.01±0.697	1.41±3.809	2.08±0.600	2.36±3.643	3.35±0.918	0.51±0.446	2.87±0.669	1.79±4.495
Grade 9 (*N* = 28)	3.27±1.301	0.71±0.338	1.87±0.557	0.82±0.572	2.92±0.736	0.33±0.449	2.64±0.820	3.52±5.656

https://figshare.com/articles/figure/tabel1-4/19149803?file=34023134

The results for sensitivity *d´* showed that the grade main effect was not significant; the gender main effect was significant, *F*_(1,120)_ = 4.201, *p*<0.05, *η*^2^ = 0.037, with boys less sensitive than girls; the sensation seeking main effect was significant, *F*_(1,120)_ = 15.701, *p*<0.001, *η*^2^ = 0.127, with high sensation seeking adolescents less sensitive than low sensation seeking adolescents. The interaction between grade and gender was not significant; the interaction between grade and sensation seeking was not significant; the interaction between gender and sensation seeking was significant, *F*_(1, 120)_ = 4.794, *p*<0.05, *η*^2^ = 0.042. High sensation seeking boys are less sensitive than low sensation seeking boys, but there is no significant difference in sensitivity among girls with different sensation seeking levels; the interaction between grade, gender and sensation seeking was not significant.

The results of the judgment criterion *β* showed that the main effect of grade was not significant; the main effect of gender was not significant; the main effect of sensation seeking was significant, *F*_(1,120)_ = 6.328, *p*<0.05, *η*^2^ = 0.055, and the judgment criterion for high sensation seeking adolescents was higher than that for low sensation seeking adolescents; the interaction of grade and gender was not significant; the interaction of grade and sensation seeking was not significant; the interaction of gender and sensation seeking was not significant; the interaction of grade, gender and sensation seeking was not significant.

## General discussion

Based on the two-stage processing model of HP, this study established a relationship between adolescents’ sensation seeking level and HP ability, and explored the role of sensation seeking in both perception stage and behaviour response stage.

### The interaction of grade, gender and sensation seeking in the stage of perception in adolescents

During traffic road HP, latency of first fixation is the time it takes for a subject to see a specific area of interest from the start of the presentation of a video, and is one of the temporally important reference data in eye-tracking metrics [20], which marks the speed at which individuals identify hazards on the road. The results showed that there was no significant difference in latency of first fixation among adolescent girls with different sensation seeking levels, but low sensation seeking boys had a longer latency of first fixation than high sensation seeking boys. This may be because sensation seeking is associated with novel stimuli [21], and once a hazard is present on a traffic road, high sensation seeking boys are the first to catch the novel change and are able to perceive the change on the road more quickly and recognise the hazard. Previous researchers have suggested that adolescent boys have higher levels of sensation seeking than girls and that girls slowly develop a preference for choosing safety [22], so there is no significant difference in how quickly girls with different levels of sensation seeking recognise danger, while high sensation seeking boys are more aware of danger than low sensation seeking boys.

The duration of first fixation is the duration of the first fixation point that appears in an individual’s AOI, and in traffic road HP, the duration of first fixation is characterized by the degree of attention paid to a fixed area [23]. The results showed that in 7^th^ grade, there was no significant difference in the duration of first fixation between sensation seeking groups for either boys and girls; in 8^th^ grade, high sensation seeking boys began to perceive hazards longer than low sensation seeking boys, and there was no significant difference in the duration of perception stage between sensation seeking girls; in 9^th^ grade, high sensation seeking boys had a longer duration of first fixation than low sensation seeking boys, but high sensation seeking girls had a shorter duration of the first fixation than low sensation seeking girls. The fact that sensation seeking becomes a major factor in the duration of first fixation from 8^th^ grade may be due to the "maximum at 8^th^ grade" phenomenon in the personality trait of sensation seeking [24]. The social-emotional system of adolescents in adolescence is developing and prefers a state of high emotional arousal [14], so not only do high sensation seeking adolescents recognize danger quickly, but boys are more likely to be attracted to novel stimuli, which is why high sensation seeking boys in 8^th^ and 9^th^ grades have a longer early processing time of danger than low sensation seeking boys. However, the present study also found that the early processing time of danger became longer for girls at the low sensation seeking level in 9^th^ grade instead, which may be an evolutionary basis for the difference between boys and girls, with girls tending to have superior processing of fearful stimuli than boys [25], and girls being more sensitive to dangerous stimuli, having self-protective mechanisms and a more cautious, so low sensation seeking girls have a bias and advantage for attentional processing of negative stimuli [26].

### The interaction of gender and sensation seeking in the stage of behaviour response in adolescents

In signal detection theory, sensitivity refers to the degree to which one can quickly discriminate where a signal is and identify danger among a lot of noise; the higher the value, the better the discrimination of danger, and vice versa [27]. The present study found no significant difference in the higher sensitivity of girls across sensation seeking levels, possibly because dangerous videos are emotional stimulus signals and girls activate more brain areas during emotional recognition [28]. Girls have an advantage over boys in recognising emotional information viewed from videos [29], and have more sustained contemplation of novel stimuli [30], a gender difference that begins in adolescence [31]. It is also possible that the video material was a low-risk situation and that girls were more sensitive to low-risk situations [32]. This study also found that high sensation seeking boys were less sensitive than low sensation seeking boys, which may be because high sensation seeking boys tend to be stimulus seeking and have developed tolerance to certain intensity stimuli. Through the interaction test, we can see that the effect of sensation seeking on the response to risky behaviours is different for different genders of junior high school students, and the effect of sensation seeking on the response to risky behaviours is greater for boys. The gender difference between males and females may be due to the needs of psychological mechanisms that evolved during the hunting period of ancient humans, when males are responsible for hunting outside and face more dangers. Only successful hunting can obtain the capital for human reproduction. As a result, sensation seeking has evolved to be more adaptive for males, thus high sensation seeking males may have developed psychological mechanisms that underestimate danger to a certain extent and are less able to aware of it. Here it is important to note the distinction between sensitivity in the response stage of risky behaviours and danger recognition in the process of danger perception as different indicators of different stages.

Judgement criteria are rules that differentiate between the two, representing the decision criteria or response bias towards signals and noises. The smaller the value, the looser judgement criteria and vice versa [7]. This study found that although high sensation seeking adolescents were able to detect unusual movements on the traffic road more quickly, they might not define them as dangerous or may perceive them as less dangerous because of their higher criteria for defining danger, so they would act to cross road. The reason why the low sensation seeking adolescents has looser judgement criteria may be that they are more cautious when crossing road, they prefer to be more conservative and are more likely to judge traffic situations as dangerous, thus higher possibilities for them to choose not to cross the road.

In summary, this study found a clear processing advantage in danger perception for high sensation seeking junior high school boys, but this danger recognition advantage disappeared during the behavioural response stage due to their weaker sensitivity *d´* and stricter judgement criteria *β*. Some researchers have used a dual-system model to explain adolescent risk-taking behaviours [22], suggesting that two systems in the adolescent brain: the socio-emotional system and the cognitive control system have matured during junior high school to enhance their pursuit of stimuli, novelty and risky behaviours, and that the slower-maturing cognitive control system has not yet developed to adequate to suppress these risky impulses at the appropriate level [33]. This study argues that risky behaviour in road crossing is a product of high sensation seeking and low impulse control, and that high sensation seeking junior high school boys have a clear advantage in the danger perception stage, but that the attraction of high emotional arousal causes them to become desensitised to danger and raises the criteria of danger judgement, seeing possible danger but not paying enough attention to it, thus reversing the perceived advantage into risky behaviour and producing the "looked but failed to see" effect. The personality trait of sensation seeking can become the internal cause of explicit dangerous behaviour, which reveals the regulatory role of personality factors behind adolescents’ hazard behaviour. This study provides new evidence for the relationship between sensation seeking and HP.

### Countermeasures for improving HP in adolescents

This study also provides a direction for adolescents’ traffic and road safety training. The results of this study suggest that boys begin to show ’looked but failed to see’ to danger after 8^th^ grade and should therefore be the focus of danger perception interventions in this stage. For example, the State Perception Assessment System [34] was used to train junior high school students in hazard perception, asking "What is danger?" while watching a video on danger, "Where is the danger?" "What will happen next?" This reinforces their perception of danger. At the same time, awareness of rules and laws should be cultivated, for example, by using real traffic accidents in the region as case studies to discuss the background, causes and responsibilities of traffic accidents, and to learn how to avoid risks. Thirdly, it is also important to combine safety education with practical exercises. For example, in Japan schools, guardians are asked to accompany students on "school walks" several times, discuss possible risk points and dangers on the road and encourage students to suggest ways to avoid them. Finally, it is important to inspire a sense of social responsibility in adolescents, to reduce the impulsiveness of high sensation seeking boys and to improve their self-control capabilities.

### Limitations and deficiencies

Firstly, the subjects of this study are all from junior high schools in Dalian, and the scope is relatively limited. There are no subjects from other regions, nor from villages or urban-rural fringe. The sample is relatively single. Particularly, the effect range of some key results were sort of small, which may be due to the large differences among individuals in the group and the relatively large variance among indiviuals. In future research, other scales and measurement methods are needed to explore the impact of other personality factors on HP, control irrelevant variables as much as possible and improve the experiment.

Secondly, the video materials used in this study can be improved in definition and shooting angle. The subjects have Hawthorne effect. Adolescents will pretend to make good performance in order to get praise and recognition from teachers. If virtual technology or experiments can be carried out in real scenes, the psychological stimuli received by the subjects will be more real, the reaction will also be more realistic, making the experimental data more convincing.

## Conclusion

This study demonstrates that high sensation seeking junior school boys’ perception of road hazards is "looked but failed to see", specifically that high sensation seeking boys perceive hazards faster than girls and low sensation seeking boys during the perception stage, that they pick up novel stimuli more quickly, and that from the 8^th^ grade onwards, in the early stages of perception processing, they showed a greater attentional processing advantage for hazard. However, this advantage becomes a disadvantage in the behaviour response stage, where they are less sensitive than in behaviour response stage, have stricter judgement criteria, do not treat potential hazards as hazards or perceive them as less dangerous, and are more likely to make dangerous decisions when crossing road.

## Supporting information

S1 File Appendix A. (DOCX)

S2 File Appendix A in Chinese. (DOCX)

S3 File Appendix B. (DOCX)

S4 File Appendix B in Chinese. (DOCX)

S5 File Appendix C. (DOCX)

S6 File Appendix C in Chinese. (DOCX)

S7 File Data research 1. (XLS)

S8 File Data research 2. (XLS)

## References

[1] Road traffic safety research center of the Ministry of public security, Disease Control and Prevention Center of Chinese, Center of chronic non communicable diseases(CHN). The Research Report on the Status of Road Traffic Injuries among Children in China.People’s Health Publishing House:China,2014.

[2] The State Council.Notice on the outline for the development of Chinese women and children.[cited 2021 April 23].Available from: http://www.gov.cn/zhengce/content/2021-09/27/content_5639412.htm.

[3] Tapiro H, Oron-Gilad T, Parmet Y. The effect of environmental distractions on child pedestrian’s crossing behavior. Safety sci.,2018, 106:219–229. doi: 10.1016/j.ssci.2018.03.024 .32199553

[4] Rosenbloom T, Mandel R, Rosner Y, Eldror E. Hazard perception test for pedestrians. Accid.Anal.Prev. 2015,79,160–169. doi: 10.1016/j.aap.2015.03.019 . 25838190

[5] Castanier C, Paran F, Delhomme P. Risk of crashing with a tram: perceptions of pedestrians, cyclists,and motorists.Transport.Res.Part F. 2012,15,387–394. doi: 10.1016/j.trf.2012.03.001

[6] Sun L, Chang RS. Review of Research on Drivers’Hazard Perceptiondoi. J.Psychol.sci.2014,37(6), 1354–1358. doi: 10.16719/j.cnki.1671-6981.2014.06.012

[7] Egea-Caparrós DA, García-Sevilla J, Pedraja MJ, Romero-Medina A, Marco-Cramer M, Pineda-Egea L. Late detection of hazards in traffic: A matter of response bias? Accid. Anal. Prev.2016,94:188–197. doi: 10.1016/j.aap.2016.06.002 . 27328018

[8] Rosenbloom T, Wolf Y. Sensation seeking and detection of risky road signals: a developmental perspective. Accid. Anal.Prev.2002,34,569–580. doi: 10.1016/s0001-4575(01)00054-9 . 12214951

[9] Alonso F, Esteban C, Useche S, Colomer N. Effect of road safety education on road risky behaviors of Spanish children and adolescents:findings from a National Study. Int J Environ Res Public Health.2018,15(12):2828. doi: 10.3390/ijerph15122828 . 30545039 PMC6313808

[10] Demetre JD, Lee DN, Pitcairn TK, Grieve R, Thomson JA. Ampofo-Boateng K. Errors in young children’s decisions about traffic gaps: experiments with roadside simulations.Brit. J.Psychol.1992, 83,189–202. doi: 10.1111/j.2044-8295.1992.tb02434.x . 1611407

[11] Meir A, Oron-Gilad T, Parmet Y. Are child-pedestrians able to identify hazardous traffic situations?Measuring their abilities in a virtual reality environment. Saf. Sci.2015,80, 33–40. doi: 10.1016/j.ssci.2015.07.007

[12] O’Neal EE, Plumert JM. How do parents teach children to cross roads safely? A study of parent-child road-crossing in an immersive pedestrian simulator. Inj. Prev. 2018,24:A17–A18. doi: 10.1136/injuryprevention-2018-safety.48

[13] Wang HR, Shi LC, Miao LQ, Tan DL. The Development of Pupil’s Visual Search in Traffic Situation. J.Psychol.sci.2018,41(3),601–607. doi: 10.16719/j.cnki.1671-6981.20180314

[14] Collado A, Felton JW, MacPherson L, Lejuez C.W. Longitudinal trajectories of sensation seeking, risk taking propensity,and impulsivity across early to middle adolescence. Addict. Behav.2014,39,1580–1588. doi: 10.1016/j.addbeh.2014.01.024 . 24566195 PMC4117709

[15] Koekemoer K, Gesselleen MV, Niekerk AV, Govender R, As ABV. Child pedestrian safety knowledge, behaviour and road injury in cape town,south Africa.Africa. Accid. Anal. Prev. 2017,99,202–209. doi: 10.1016/j.aap.2016.11.020 . 27960100

[16] Wang H, Shi L, Schwebel DC. Relations between adolescent sensation seeking and traffic injury: Multiple-mediating effects of road safety attitudes, intentions and behaviors. Traffic. Ini. Prev. 2019,20(8),789–795. doi: 10.1080/15389588.2019.1666982 . 31738575 PMC10413049

[17] Du XY, Ma JF, Chang RS. The interactive effect of vehicle signals and sensation-seeking on driver hazard perception.Transport Res.F.2020,73,174–187. doi: 10.1016/j.trf.2020.06.018

[18] Duell N, Steinberg L, Chein J, AlHassan SM, Bacchini D, Lei C, et al. Interaction of reward seeking and self-regulation in the prediction of risk taking:a cross-national test of the dual systems model. Dev. Psychol. 2016,52(10), 1593–1605. doi: 10.1037/dev0000152 . 27598251

[19] Sagberg F, Bjørnskau T. (2006). Hazard perception and driving experience among novice drivers. Accid.Anal.Prev, 38(2), 407–414. doi: 10.1016/j.aap.2005.10.014 . 16313881

[20] Yang MM. Eye Tracking and Chinese Reading. Social science literature press:BeiJing, China,2018; p.44–65.

[21] Steinberg L. A social neuroscience perspective on adolescent risk-taking. Dev.Rev.2008,28(1), 78–106. doi: 10.1016/j.dr.2007.08.002 . 18509515 PMC2396566

[22] Steinberg L, Albert D, Cauffman E, Banich M, Graham S, Woolard.Age differences in sensation seeking and impulsivity as indexed by behavior and self-report:evidence for a dual systems model. Dev Psychol.2008, 11;44(6):1764–78. doi: 10.1037/a0012955 . 18999337

[23] Wang HR. Application and Prospect of eye movement technology in road traffic research Med J of Communications.2014, 28(02):119–123+126.

[24] Hu CHM, Zhang XY, He HM. The development characteristics of teenagers’ sensation seeking and impulsivity.Mental Health Education In. Schools.2018,16:20–24.

[25] McClure EB, Monk CS, Nelson EE, Zarahn E, Leibenluft E, Bilder RM, et al. A developmental examination of gender differences in brain engagement during evaluation of threat.Biol Psychiat.2004, 55(11),1047–1055. doi: 10.1016/j.biopsych.2004.02.013 . 15158422

[26] Kang Q LI H. The Effect of Gender Difference on Fear Stimuli Processing Under Implicit Paradigm. Journal of Southwest China Normal U niversity (Natural Science Edition). 2011(03),70–73. doi: 10.13718/j.cnki.xsxb.2011.03.030

[27] Wallis TSA, Horswill MS. Using fuzzy signal detection theory to determine why experienced and trained drivers respond faster than novices in a hazard perception test. Accid.Anal.Prev.2007,39(6), 1177–1185. doi: 10.1016/j.aap.2007.03.003 . 17920841

[28] Hofer A, Siedentopf CM, Ischebeck A, Rettenbacher MA, Fleischhacker WW. Gender differences in regional cerebral activity during the perception of emotion: a functional MRI study.NeuroImage. 2006.32(2),854–862. doi: 10.1016/j.neuroimage.2006.03.053 . 16713306

[29] Wingenbach TS, Ashwin C, Brosnan M. Sex differences in facial emotion recognition across varying expression intensity levels from videos. PLoS One.2018,13(1),e0190634. doi: 10.1371/journal.pone.0190634 . 29293674 PMC5749848

[30] Xu S, Yuan JJ, Lu H, Pan Y, Zhao SY, Luo Y, et al.The Female Bias in Processing Novel Events: An ERP Study.2016,J.Ps chol.sci.39(03):666–672. doi: 10.16719/j.cnki.1671-6981.20160323

[31] Lee NC, Krabbendam L, White TP, Meeter M, Banaschewski T, Barker GJ. Do you see what I see? Sex differences in the discrimination of facial emotions during adolescence.Emotion, 2013,13(6):1030–40. doi: 10.1037/a0033560 . 23914763

[32] Zhu P, Chang RS. Effects of Gender and Situational Hazard Level on Pedestrian Hazard Perception:Evidence from ERP.Studies Physiol.Behav.2020,18(01):113–120. 10.1016/j.neulet.2019.134546 31629775

[33] Shulman EP, Smith AR, Silva K, Icenogle G, Duell N, Chein J, et al. The dual systems model:review,reappraisal,and reaffirmation. Dev. Cogn. Neurosci. 2016.17,103–117. doi: 10.1016/j.dcn.2015.12.010 . 26774291 PMC6990093

[34] Ventsislavova P, Gugliotta A, Peña -Suarez E, Garcia-Fernandez P, Eisman E, Crundall D, et al. What happens when drivers face hazards on the road? Accid.Anal. Prev.2016,91,43–54. doi: 10.1016/j.aap.2016.02.013 . 26954761

