# Supplementary material for: An analysis of the adolescents’ hazard perception when crossing road from the perspective of personality characteristics based on an eye-tracking study
Source: PLoS One. 2022 May 6;17(5):e0267309. doi: 10.1371/journal.pone.0267309 (PMC9075635; doi:10.1371/journal.pone.0267309)
Supplement: S1 File — (DOCX) [file pone.0267309.s001.docx]

Appendix A

Sensation seeking questionnaire for primary and secondary schools

Instruction: Hello, classmate! This is a questionnaire to investigate your interests and hobbies. There is no good or bad answer. Please choose from the alternative answers according to your real thoughts.

| Question number | title | Don't want to do | Want to do it, but have to do it | want to do it, if have a chance, must do it |
| --- | --- | --- | --- | --- |
| （1） | Skydiving | 1 | 2 | 3 |
| （2） | Surfing | 1 | 2 | 3 |
| （3） | Tattoo | 1 | 2 | 3 |
| （4） | Bungee jumpin | 1 | 2 | 3 |
| （5） | High altitude springboard | 1 | 2 | 3 |
| （6） | Trek the wilderness | 1 | 2 | 3 |
| （7） | Drunk | 1 | 2 | 3 |
| （8） | Harass others | 1 | 2 | 3 |
| （9） | Do something against the rules | 1 | 2 | 3 |
| （10） | Cave exploration | 1 | 2 | 3 |
| （11） | Sail far by sailboat | 1 | 2 | 3 |
| （12） | Diving through reefs at the bottom of the sea | 1 | 2 | 3 |
| （13） | Climb a steep mountain | 1 | 2 | 3 |
| （14） | Take pleasure in other people's misfortune | 1 | 2 | 3 |
| （15） | With wild people | 1 | 2 | 3 |
| （16） | Diving in a diving suit | 1 | 2 | 3 |
| （17） | Talk dirty with friends | 1 | 2 | 3 |
| （18） | Date someone sexy and attractive | 1 | 2 | 3 |
| （19） | Be rude to teachers and elders | 1 | 2 | 3 |
| （20） | Meet gay people | 1 | 2 | 3 |
| （21） | Gallop on horseback | 1 | 2 | 3 |
| （22） | Racing | 1 | 2 | 3 |
| （23） | Attend a carnival | 1 | 2 | 3 |
| （24） | Steal by chance | 1 | 2 | 3 |
| （25） | Skiing on steep snow mountains | 1 | 2 | 3 |
| （26） | Associate with elusive people | 1 | 2 | 3 |
| （27） | Travel by jet | 1 | 2 | 3 |
| （28） | Go to sea alone in a boat | 1 | 2 | 3 |
| （29） | Reckless gambling | 1 | 2 | 3 |
| （30） | Watch an accident | 1 | 2 | 3 |
