# Supplementary material for: An analysis of the adolescents’ hazard perception when crossing road from the perspective of personality characteristics based on an eye-tracking study
Source: PLoS One. 2022 May 6;17(5):e0267309. doi: 10.1371/journal.pone.0267309 (PMC9075635; doi:10.1371/journal.pone.0267309)
Supplement: S2 File — (DOCX) [file pone.0267309.s002.docx]

# 附录A 中小学感觉寻求问卷

指导语：同学你好！这是一份调查兴趣爱好的问卷，答案没有好坏之分，请根据你的真实想法在备选答案中进行选择。

| 题号 | 题 目 | 不想做 | 想做，但不一定去做 | 想做，若有机会一定去做 |
| --- | --- | --- | --- | --- |
| （1） | 跳伞 | 1 | 2 | 3 |
| （2） | 冲浪 | 1 | 2 | 3 |
| （3） | 刺青或纹身 | 1 | 2 | 3 |
| （4） | 蹦极 | 1 | 2 | 3 |
| （5） | 高空跳板 | 1 | 2 | 3 |
| （6） | 跋涉荒野 | 1 | 2 | 3 |
| （7） | 醉酒 | 1 | 2 | 3 |
| （8） | 骚扰别人 | 1 | 2 | 3 |
| （9） | 做违反常规的事 | 1 | 2 | 3 |
| （10） | 穿山洞探险 | 1 | 2 | 3 |
| （11） | 乘帆船远航 | 1 | 2 | 3 |
| （12） | 潜水时在海底穿过暗礁 | 1 | 2 | 3 |
| （13） | 爬陡峭的山 | 1 | 2 | 3 |
| （14） | 幸灾乐祸 | 1 | 2 | 3 |
| （15） | 与放荡不羁的人在一起 | 1 | 2 | 3 |
| （16） | 穿潜水衣潜水 | 1 | 2 | 3 |
| （17） | 与朋友讲下流话 | 1 | 2 | 3 |
| （18） | 与性感有魅力的人约会 | 1 | 2 | 3 |
| （19） | 对老师与长辈无礼 | 1 | 2 | 3 |
| （20） | 与同性恋者结识 | 1 | 2 | 3 |
| （21） | 骑马疾驰 | 1 | 2 | 3 |
| （22） | 赛车 | 1 | 2 | 3 |
| （23） | 参加狂欢饮酒会 | 1 | 2 | 3 |
| （24） | 凭侥幸心理偷窃 | 1 | 2 | 3 |
| （25） | 在险峻的雪山上滑雪 | 1 | 2 | 3 |
| （26） | 和难以捉摸的人交往 | 1 | 2 | 3 |
| （27） | 乘喷气式飞机旅游 | 1 | 2 | 3 |
| （28） | 乘小舟只身出海 | 1 | 2 | 3 |
| （29） | 不顾后果的赌博 | 1 | 2 | 3 |
| （30） | 看一场意外车祸 | 1 | 2 | 3 |
