# Supplementary material for: An analysis of the adolescents’ hazard perception when crossing road from the perspective of personality characteristics based on an eye-tracking study
Source: PLoS One. 2022 May 6;17(5):e0267309. doi: 10.1371/journal.pone.0267309 (PMC9075635; doi:10.1371/journal.pone.0267309)
Supplement: S3 File — (DOCX) [file pone.0267309.s003.docx]

Appendix B

Traffic content and duration in video

| Video sequence | Video duration (*s*) | Zebra crossing | Traffic lights | Dangerous location | Description of hazards |
| --- | --- | --- | --- | --- | --- |
| 1 | 12 | Yes | Yes | Far | The motor vehicle turns behind the motor vehicle waiting for a red light on the road and enters the road |
| 2 | 5 | Yes | Yes | Near | The motor vehicle turns behind the pedestrian on the right and enters the road |
| 3 | 12 | No | No | Near | The motor vehicle turns right into the road, and the electric pole affects the pedestrian's sight |
| 4 | 9 | No | No | Far | The motor vehicle comes from a distance, and other vehicles park on the roadside affect the sight of pedestrians |
| 5 | 12 | Yes | Yes | Near | The motor vehicle starts after the traffic light changes |
| 6 | 9 | No | No | Far | The motor vehicle comes from a distance, roadside parked vehicles and roadside trees affect the sight of pedestrians |
| 7 | 9 | Yes | No | Far | The motor vehicle turns on the turn signal in advance and turns left to enter the road |
| 8 | 9 | No | Yes | Far | The motor vehicle turns right into the road without the turn signal |
| 9 | 10 | No | No | Near | The motor vehicle turns right into the road without the turn signal |
| 10 | 9 | No | No | Far | The motor vehicle turns right into the road, and the vehicles parked on the roadside affect the pedestrian's sight |
| 11 | 12 | No | No | Far | When the motor vehicle turns left and enters the road, the motor vehicle on the road affects the pedestrian's sight |
| 12 | 10 | Yes | No | Far | The motor vehicle turns right into the road, and the curved road affects the pedestrian's sight |
