# Supplementary material for: An analysis of the adolescents’ hazard perception when crossing road from the perspective of personality characteristics based on an eye-tracking study
Source: PLoS One. 2022 May 6;17(5):e0267309. doi: 10.1371/journal.pone.0267309 (PMC9075635; doi:10.1371/journal.pone.0267309)
Supplement: S4 File — (DOCX) [file pone.0267309.s004.docx]

附录1

视频中的交通内容及视频时长
