# Supplementary material for: An analysis of the adolescents’ hazard perception when crossing road from the perspective of personality characteristics based on an eye-tracking study
Source: PLoS One. 2022 May 6;17(5):e0267309. doi: 10.1371/journal.pone.0267309 (PMC9075635; doi:10.1371/journal.pone.0267309)
Supplement: S5 File — (DOCX) [file pone.0267309.s005.docx]

Appendix C

traffic content and duration in video

| Video sequence | Video duration (s) | Zebra crossing | Traffic lights | Whether there is danger (if there is danger, describe the danger) |
| --- | --- | --- | --- | --- |
| 1 | 11 | Yes | Yes | Yes（When the motor vehicle turns left and enters the road, the pedestrian traffic light is green） |
| 2 | 6 | Yes | Yes | No |
| 3 | 9 | Yes | Yes | Yes（When the motor vehicle turns right into the road, the pedestrian traffic light is red） |
| 4 | 8 | Yes | Yes | No |
| 5 | 11 | No | No | No |
| 6 | 12 | No | No | Yes（The motor vehicle drives into the road from a distance, and street trees affect the sight of pedestrians） |
| 7 | 10 | No | No | Yes（The motor vehicle enters the road from a distance, curves and roadside trees affect the sight of pedestrians） |
| 8 | 10 | No | No | No |
| 9 | 10 | Yes | Yes | No |
| 10 | 10 | Yes | Yes | No |
| 11 | 13 | Yes | Yes | Yes（The traffic lights change and the motor vehicle starts） |
| 12 | 10 | Yes | Yes | No |
| 13 | 12 | No | Yes | Yes（The motor vehicle drives into the road from a distance） |
| 14 | 10 | No | No | Yes（The motor vehicle turns left and enters the road without turning on the turn signal） |
| 15 | 11 | Yes | Yes | No |
| 16 | 10 | Yes | No | No |
| 17 | 10 | No | No | Yes（The motor vehicle drives into the road from a distance, vehicles parked on the roadside and roadside trees affect the sight of pedestrians） |
| 18 | 10 | No | No | No |
| 19 | 9 | No | No | Yes（A motor vehicle comes from a nearby place, vehicles parked on the roadside affect the sight of pedestrians） |
| 20 | 12 | No | No | Yes（The motor vehicle turns right and drives into the road from a distance, vehicles parked on the roadside affect the sight of pedestrians） |
